# Recurrence of cervical cancer and its resistance to progestin therapy in a mouse model

**DOI:** 10.18632/oncotarget.13676

**Published:** 2016-11-29

**Authors:** Fabiola F Mehta, Seunghan Baik, Sang-Hyuk Chung

**Affiliations:** ^1^ Center for Nuclear Receptors and Cell Signaling, Department of Biology and Biochemistry, University of Houston, Houston, TX 77204, USA

**Keywords:** cervical cancer, recurrence, therapy resistance, medroxyprogesterone acetate (MPA), human papillomavirus (HPV)

## Abstract

Studies using *K14E6/K14E7* transgenic mice expressing E6 and E7 oncoprotein of human papillomavirus type 16 (HPV16) have demonstrated that estrogen (E_2_) is required for the genesis and growth of cervical cancer. Our prior study using the same mouse model has showed that progestin drug medroxyprogesterone acetate (MPA) promotes regression of primary cervical cancer. In the present study, we use the same transgenic mouse model to determine whether the cancer recurs after MPA therapy. Cervical cancer recurred even if MPA treatment was continued. Unlike primary cervical cancer, the cancer recurred even in the absence of exogenous E_2_ when MPA treatment was ceased. Furthermore, recurrent cervical cancer did not fully regress upon MPA treatment. Our results support that MPA fails to completely eliminate primary cervical cancer cells and that remaining cancer cells grow independent of exogenous E_2_ and are refractory to MPA.

## INTRODUCTION

High−risk human papillomaviruses (HPVs) are causally associated with various human cancers, among which cervical cancer is most notable [1]. E6 and E7 viral oncoproteins are primarily responsible for the tumorigenic potential of these viruses. Among many cellular proteins interacting with these viral oncoproteins, p53 and pRb tumor suppressor are the most prominent target of E6 and E7, respectively [2]. Among more than a dozen high−risk HPVs, HPV16 is most commonly found in cancer, followed by HPV18 [1]. Worldwide, cervical cancer is the fourth most common cancer and the fourth leading cause of death by cancer in women [3]. The Pap test and prophylactic HPV vaccines are effective in preventing cervical cancer [4]. However, they are not readily available to most women in developing countries and women of low socio−economic status in some developed countries [5]. Current therapies for advanced or recurrent cervical cancer are not effective [6]. The development of effective therapy for cervical cancer is urgently needed.

While most women are infected with high−risk HPVs during their lifetime, only a small fraction of women succumb to cervical cancer, and precancerous lesion called cervical intraepithelial neoplasia (CIN) often regresses spontaneously [7]. These observations suggest that HPV is not sufficient and other cofactors are required for cervical carcinogenesis. In HPV−infected women who have used oral contraceptives compared to those who have not, the risk of developing cervical cancer is increased in proportion to the duration of use [8]. In HPV−infected women, the risk for cervical cancer in women with multiple full−term pregnancies is significantly higher compared to nulliparous women [9]. These observations suggest that female sex hormones, estrogen (E_2_) and progesterone (P_4_) may play a role in HPV−induced cervical cancer. Epidemiological studies looking at individual roles of these hormones in cervical cancer, however, have been inconclusive mainly due to the lack of data stratification based on the status of high−risk HPV and low cervical cancer incidence in the study population (i.e., post−menopausal women). A few clinical trials have not been informative due to short follow−up period, poor drug choice, and/or underpowered multivariable analyses. These studies are discussed in other publications [10, 11].

Transgenic mouse models expressing HPV16 E6 and/or E7 have been powerful tools to understand the molecular mechanism of cervical carcinogenesis. In these mice, expression of E6 and E7 is targeted to the squamous epithelium, which is the natural target for productive HPV infection [12–14]. Consistent with the notion that HPV is not sufficient, the development of cervical cancer in these mice requires both HPV oncogenes and chronic treatment with low levels of exogenous E_2_ [12, 15]. Cervical cancer arising in the HPV transgenic mouse model recapitulates key aspects of the human cancer including cofactor−dependent progressive disease development, cancer development in the transformation zone, and expression of similar biomarkers [12, 15, 16]. Estrogen receptor α (ERα) is required for cervical carcinogenesis in the HPV transgenic mice [17, 18]. One of ERα target genes in the cervix is *Pgr* coding for progesterone receptor (PR) [19]. ERα and PR are ligand−dependent transcription factors belonging to the nuclear receptor superfamily [20]. PR−positive cervical cancer patients have better prognosis after radiation therapy than PR−negative cancer patients [21]. Although it needs to be confirmed by independent studies, the use of progestin drug medroxyprogesterone acetate (MPA) is inversely associated with cervical neoplastic disease in HPV−infected women [22]. P_4_ inhibits E_2_−induced cell proliferation and promotes apoptosis in the murine cervical epithelium in the PR−dependent manner [19]. While PR is expressed in the HPV transgenic mouse model for cervical cancer, its activity is minimal because P_4_ levels are kept low [10, 23]. These observations suggest that PR may be a ligand−dependent tumor suppressor in cervical cancer. In agreement, MPA promotes regression of cervical cancer in the HPV transgenic mouse model [23]. In the present study, we demonstrate that cervical cancer recurs after MPA therapy. We also show that, although PR is expressed, the recurring cancer is refractory to MPA.

## RESULTS

### Cervical cancer recurs after MPA treatment is ceased

**Figure 1:**
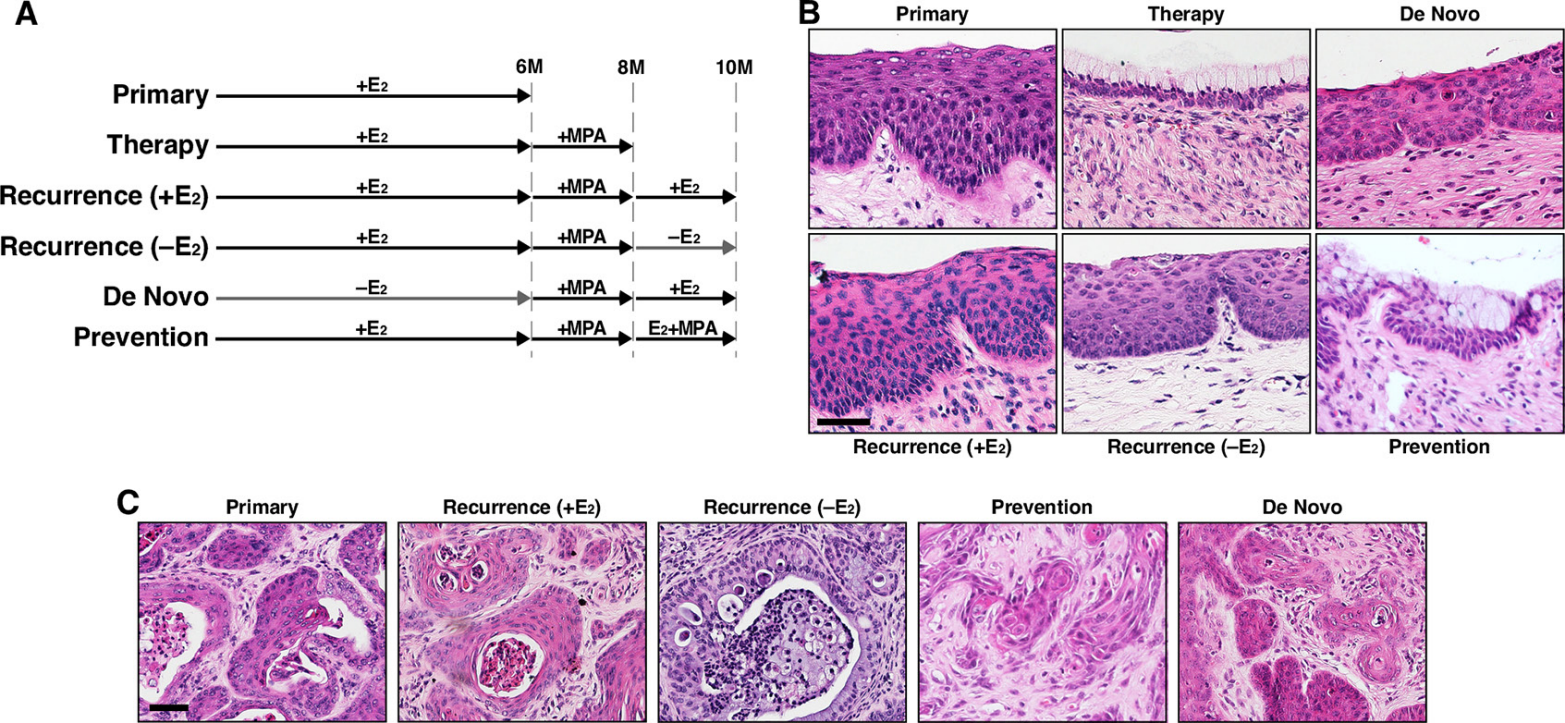
Cervical cancer recurs after MPA therapy (**A**) Treatment regimens are depicted. *K14E6/K14E7* mice were enrolled at 4−6 weeks of age. MPA, medroxyprogesterone acetate; E_2_, estrogen. (**B**) Shown are representative images of H&E−stained cervical epithelium from indicated groups. Scale bar, 50 μm. (**C**) Shown are representative images of H&E−stained cervical cancer from indicated groups. Scale bar, 50 μm.

We previously showed that MPA promoted regression of cervical cancer in *K14E6/K14E7* double transgenic mice [23]. We sought to determine whether the cancer recurs after MPA therapy. *K14E6/K14E7* mice were treated with E_2_ for 6 months, and one group of mice were sacrificed immediately (primary group; Figure 1A). All seven mice in this group had cervical cancer (Table 1), indicating that all identically treated mice in other groups illustrated in Figure 1A had the cancer before further treatments. Another group of mice were subsequently treated with MPA for 2 months (therapy group; Figure 1A), and none of six mice had cervical cancer and CIN lesions (Table 1). These results indicated that cervical cancer and CIN regressed upon MPA treatment as previously demonstrated [23]. The third group was initially treated identically to the therapy group, and then retreated with E_2_ for 2 months [recurrence (+E_2_) group; Figure 1A]. All six mice in this group had cervical cancer and CIN lesions (Table 1). As a control, a group of mice were left untreated for first 6 months and then treated with MPA for 2 months followed by E_2_ treatment for 2 additional months (De Novo group; Figure 1A). Cervical cancer arising in this group was considered as new disease based on prior results that, in HPV transgenic mice expressing E6 and E7, cervical cancer did not develop without exogenous E_2_ and was regressed by MPA [12, 23]. In this group, cervical cancer developed in four of sixteen mice (25%) (Table 1), which was significantly different from cancer incidence of the recurrence (+E_2_) group (*P* = 0.002). These results indicated that most of cervical cancers in the recurrence (+E_2_) group were recurring diseases rather than newly arising cancers.

**Table 1:** Summary of worst neoplastic diseases in the lower reproductive tract of *K14E6/K14E7* mice*

Group	Group size,n	No disease	Dysplasia only			Cancer & dysplasia	Cancer incidence,%	Multiplicity (Mean ± S.E.M)
			CIN1(VaIN1)	CIN2(VaIN2)	CIN3(VaIN3)			
Primary	7	0 (0)	0 (1)	0 (4)	0 (1)	7 (1)	100 (14.3)	1.7±0.2(0.1±0.1)
Therapy	6	6 (6)	0 (0)	0 (0)	0 (0)	0 (0)	0 (0)	0 (0)
De Novo	16	4 (6)	1 (2)	5 (4)	2 (1)	4 (3)	25 (18.8)	0.8±0.4(0.3±0.1)
Recurrence (+E_2_)	6	0 (0)	0 (1)	0 (0)	0 (1)	6 (4)	100 (66.7)	3.0±0.7(2.0±0.9)
Recurrence (−E_2_)	6	0 (1)	0 (1)	0 (0)	0 (0)	6 (4)	100 (66.7)	3.0±0.4(1.3±0.5)
Prevention	7	0 (1)	0 (2)	0 (2)	0 (0)	7 (2)	100 (28.6)	2.1±0.3(0.3±0.5)
Recurrence therapy	7^#^	0 (1)	0 (0)	0 (0)	0 (1)	7 (5)	100 (71.4)	3.1±0.7(0.9±0.3)
Control	5	0 (1)	0 (0)	0 (1)	0 (0)	5 (3)	100 (60.0)	3.2±0.5(1.2±0.6)

* Mice were scored histopathologically for the worst disease present in the cervix or, in parentheses, the vagina of each mouse. CIN, cervical intraepithelial neoplasia; VaIN, vaginal intraepithelial neoplasia. *P* < 0.01 compared to the other groups. #One mouse did not have mucinified cervical epithelium, suggesting that MPA treatment did not work in that mouse. This mouse was excluded from statistical analyses.

To determine whether exogenous E_2_ is required for recurrence of cervical cancer, the recurrence (−E_2_) group was treated identically to the therapy group for the first 8 months, and then left untreated for 2 months (Figure 1A). All six mice in the group had cervical cancer (Table 1), which was significantly different from the De Novo group (*P* = 0.002). Cervical cancer and epithelia in recurrence (−E_2_) and recurrence (+E_2_) group were similar (Figure 1B–1C). The results indicated that cervical cancer recurs independent of exogenous E_2_. Vaginal cancer develops in the same mouse model, and MPA promotes its regression [23]. Vaginal cancer incidence in the recurrence groups was significantly greater than the De Novo group (*P* = 0.05), indicating that vaginal cancer also recurred after MPA therapy independent of exogenous E_2_ (Table 1).

### MPA fails to prevent recurrence of cervical cancer

We next sought to determine whether cervical cancer recurs if MPA treatment is continued. After MPA therapy, *K14E6/K14E7* double transgenic mice were co-treated with E_2_ and MPA as shown in Figure 1A (Prevention group). All seven mice in this group had cervical cancer (Table 1). Cancer incidence in this group was significantly different from the therapy group (*P* = 0.0006). More importantly, the cancer incidence (100%) was identical to the recurrence (+E_2_) group and significantly greater than De Novo group (Table 1). These results indicate that cervical cancer recurred even in the presence of MPA. While the entire cervical epithelium in the therapy group contained cells with clear cytoplasm, not all epithelia of the prevention group had such cells (Figure 1B). Similarly, in the prevention group, some cervical cancers had cells with clear cytoplasm, but most did not (Figure 1C; data not shown). The clear cytoplasm is indicative of cervical mucinification induced by MPA [19, 23].

### Exogenous E_2_ elicits larger recurrent cervical cancer

**Figure 2:**
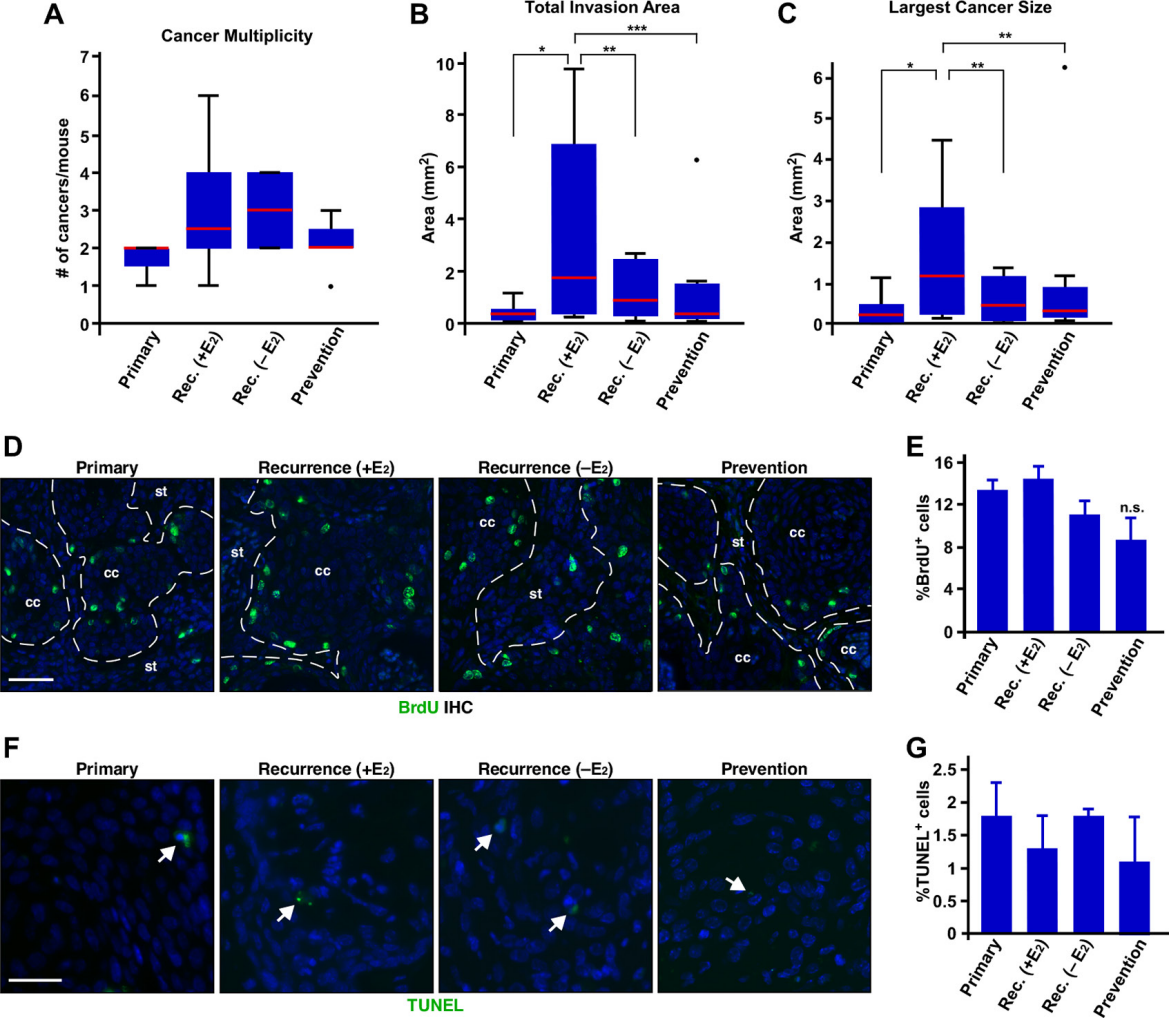
Exogenous E2 results in larger recurrent cervical cancer without affecting proliferation and apoptosis (**A**) Exogenous E_2_ does not increase the number of recurrent cervical cancer. The number of cancer in each mouse is shown as box plot. Red lines show the medians and box limits indicate the 25th and 75th percentiles. Whiskers extend 1.5 times the interquartile range. A dot indicates outlier. Group sizes are indicated in Table 1. Rec., recurrence. (**B**) Exogenous E_2_ increases total invasion area. Total invasion area per mouse is shown as box plot as described in A. Group sizes are indicated in Table 1. *P = 0.01, **P = 0.05, ***P = 0.03. (**C**) Exogenous E_2_ induces larger recurrent cervical cancer. The size of largest cancer per mouse is shown as box plot as described in A. Group sizes are indicated in Table 1. *P = 0.02, **P = 0.05. (**D**) Exogenous E2 or MPA does not affect proliferation of recurring cervical cancer cells. Cervical cancer sections were stained for BrdU (green) to measure cell proliferation. Nuclei are shown in blue. Dotted lines separate cervical cancer (cc) from stroma (st). Scale bar, 50 μm. (**E**) Results shown in D. was quantified and shown as mean ± S.E.M. (*n* = 3). (**F**) Exogenous E_2_ or MPA does not influence apoptosis of recurrent cervical cancer. Cervical cancer sections were subjected to TUNEL assay. TUNEL^+^ cells are shown in green (see white arrows). Nuclei are shown in blue. Scale bar, 30 μm. (**G**) Results shown in F. was quantified and shown as mean ± S.E.M. (*n* = 3).

While exogenous E_2_ did not affect the incidence of recurrent cervical cancer, we sought to further characterize potential role of exogenous E_2_ in recurrence of cervical cancer. Cancer multiplicity was increased in the recurrence groups compared to the primary group (Figure 2A and Table 1); however, it did not reach statistical significance (*P* = 0.07). Cancer multiplicity between recurrence (+E_2_) and recurrence (−E_2_) group was similar (Figure 2A and Table 1). Total invasion area and size of largest cancers in the recurrence (+E_2_) group were significantly larger than primary and recurrence (−E_2_) group (Figure 2B–2C). We next determined cell proliferation indices by analyzing BrdU incorporation. Percentages of BrdU−positive cells were similar among primary, recurrence (+E_2_), and recurrence (−E_2_) group (Figure 2D–2E), indicating that cancers proliferate at similar rates in all three groups. Ki67 staining showed similar results (Supplementary Figure S1A–S1B). Percentages of TUNEL−positive cervical cancer cells were similarly low in all three groups (Figure 2F–2G). We obtained similar results with cleaved caspase-3 staining (Supplementary Figure S1C–S1D). These results indicate that proliferation and apoptosis do not account for larger recurrent cervical cancer in the recurrence (+E_2_) group.

Cancer multiplicity in the prevention and recurrence (+E_2_) group was not significantly different (Figure 2A). Total invasion area and size of largest cancers in the prevention group were significantly smaller than the recurrence (+E_2_) group (*P* ≤ 0.05) (Figure 2B–2C). Proliferation indices determined by BrdU IHC was not significantly different between the recurrence (+E_2_) and prevention group (Figure 2D and 2E). We obtained similar results with Ki67 IHC (Supplementary Figure S1A–S1B). Apoptosis indices determined by TUNEL assay were also similar between the two groups (Figure 2F–2G). Cleaved caspase-3 IHC results were consistent with TUNEL assay (Supplementary Figure S1C–S1D). These results suggest that MPA delays recurrence of cervical cancer but, once recurred, MPA does not inhibit cancer growth.

### Recurrent cervical cancers are refractory to MPA

**Figure 3:**
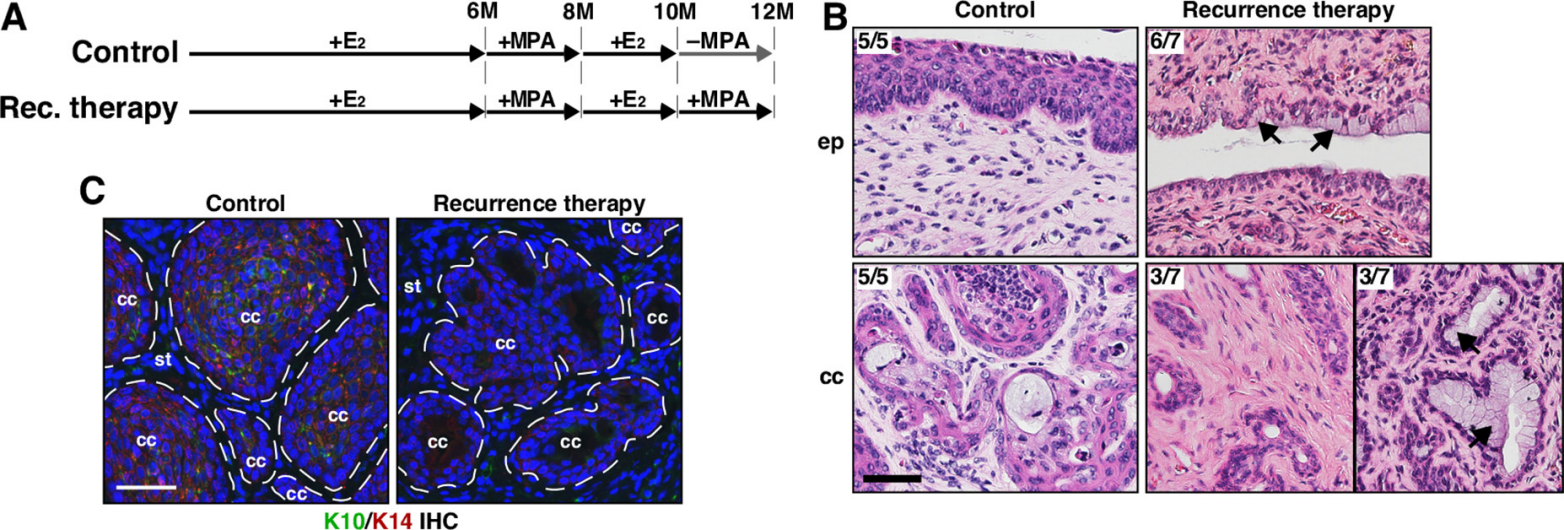
MPA fails to regress recurrent cervical cancer (**A**) Treatment regimens are shown. Mice were enrolled in the study at 4−6 weeks of age. (**B**) Recurrent cervical cancer remains after MPA therapy. Representative images of H&E−stained cervical tissue sections are shown. The number of mice with presented histology is indicated at the upper left corner. Black arrows point to cells with clear cytoplasm, indicative of mucinification. Scale bar, 50 μm. (**C**) K10 expression is decreased in recurrent cervical cancer treated with MPA. Cervical cancer sections were stained for K10 (green) and K14 (red). K14 stains cancer cells and K10 is a marker for differentiated squamous cells. Nuclei are shown in blue. Dotted lines separate cervical cancer (cc) from stroma (st). Scale bar, 50 μm.

We next sought to determine whether MPA promotes regression of recurrent cervical cancer. In order to induce recurrent cervical cancer, two additional groups of *K14E6/K14E7* double transgenic mice were treated like the recurrence (+E_2_) group and then treated with MPA (recurrence therapy group) or vehicle (control group) for 2 additional months (Figure 3A). As expected, 5 of 5 (100%) mice in the control group had cervical cancer (Table 1). In the recurrence therapy group, 7 of 7 (100%) mice had cervical cancer (Table 1). The cancer incidence in the therapy (0%) and recurrence therapy group (100%) was significantly different (*P* = 0.0006; Table 1). Mice in the recurrence therapy group had hypoplastic epithelia and epithelial cells with clear cytoplasm in the cervix (Figure 3B), indicating the functionality of MPA [23]. The cancers in the control group were well differentiated (Figure 3B). Consistently, they expressed cytokeratin 10 (K10) (Figure 3C), marker for differentiated squamous cells [24]. In the recurrence therapy group, the cancers were poorly differentiated and did not express K10 (Figure 3B–3C). Three mice had the cancer containing cells with clear cytoplasm (Figure 3B), indicative of mucinification [19]. These results indicate that MPA induces histological changes in recurrent cervical cancers, but does not eliminate them. Along with the observation that cervical cancer recurred even in the presence of MPA (see Figure 1A and Table 1), we conclude that recurrent cervical cancer is resistant to MPA. Recurrent vaginal cancer also did not regress after MPA treatment (Table 1).

### MPA shrinks recurrent cervical cancer

**Figure 4:**
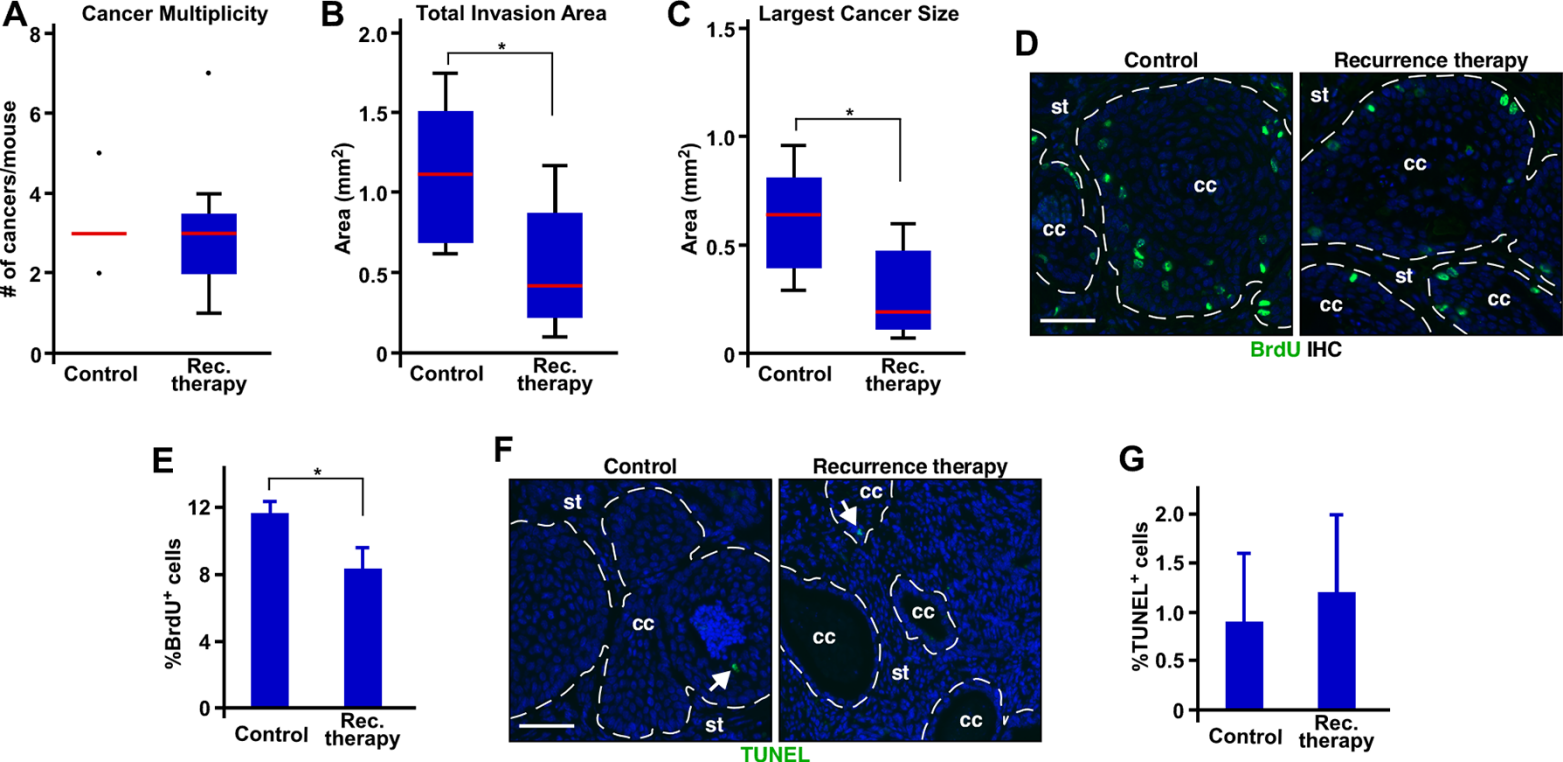
MPA decreases the size of recurrent cervical cancer by inhibiting proliferation (**A**) MPA does not decrease multiplicity of recurrent cervical cancer. The number of recurrent cervical cancer per mouse is shown in box plot. Outliers are represented by dots. Control, *n* = 5; recurrence (rec.) therapy, *n* = 6. (**B**) MPA decreases total invasion area. Total invasion area per mouse is shown in box plot. *P = 0.04 (*n* = 5 for control; *n* = 6 for recurrence therapy). (**C**) MPA decreases the size of largest cancer. Largest cancer size per mouse is shown in box plot. *P = 0.03 (*n* = 5 for control; *n* = 6 for recurrence therapy). (**D**) MPA decreases cell proliferation in recurrent cervical cancer. Cervical cancer sections were stained for BrdU (green). Nuclei are shown in blue. Dotted lines separate cancer (cc) from stroma (st). Scale bar, 50 μm. (**E**) Quantification of results shown in D. is shown as mean ± S.E.M. (*n* = 3). *P = 0.05. (**F**) MPA does not influence apoptosis of cancer cells. Cervical cancer sections were subjected to TUNEL assay. TUNEL^+^ cells are shown in green (see white arrows). Nuclei are shown in blue. Scale bar, 50 μm. (**G**) Quantification of results shown in F. is shown as mean ± S.E.M. (*n* = 3).

In agreement with the conclusion that recurrent cervical cancer is refractory to MPA, the cancer multiplicity was similar in the control and recurrence therapy group (Figure 4A and Table 1). However, total invasion area and largest cancer size were significantly smaller in the recurrence therapy group compared to the control group (Figure 4B–4C). Percentages of BrdU−positive cells were modestly, but significantly, decreased in the recurrence therapy group compared to the control group (Figure 4D–4E). Percentage of Ki67 expressing cells was also significantly decreased in the recurrence therapy group compared to the control group (Supplementary Figure S2A–S2B). Percentages of TUNEL^+^ or cleaved caspase-3^+^ cells were not significantly different between the two groups (Figure 4F–4G and Supplementary Figure S2C–S2D). These results suggest that MPA reduces the size of recurrent cervical cancer, at least in part, by inhibiting proliferation. They also indicate that recurrent cervical cancer is partially responsive to MPA.

### PR is expressed in recurrent cervical cancer

**Figure 5:**
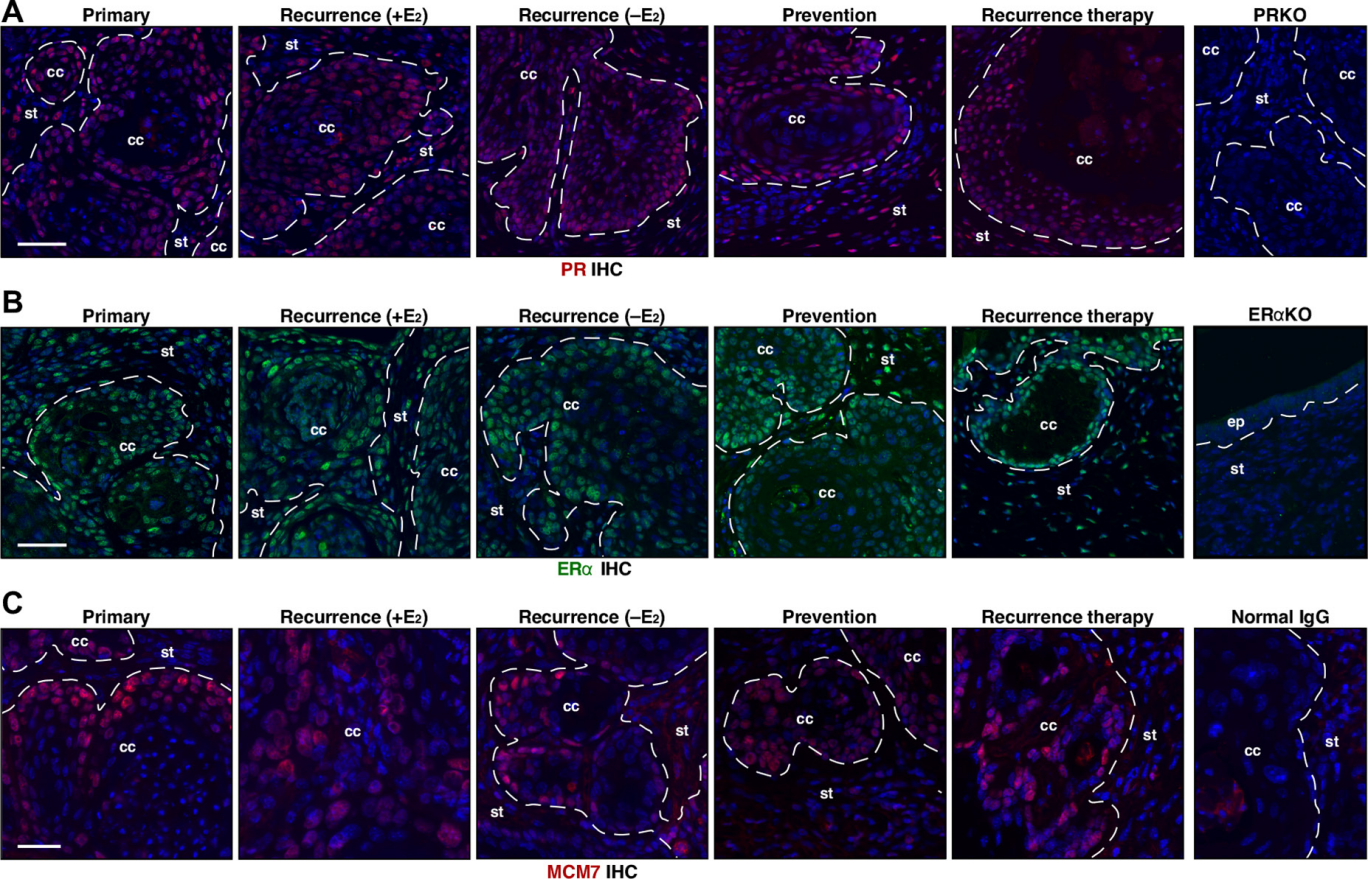
PR, ERα, and MCM7 are expressed in recurrent cervical cancer (**A**) Cancer recurrence and MPA resistance are not due to loss of PR expression. Seven cervical cancers per group were stained for PR (red) and representative images are shown. Nuclei are shown in blue. A cervical cancer section from *K14E7/Pgr*^−/−^ (PRKO) was used as negative control. Dotted lines separate cervical cancer (cc) from surrounding stroma (st). Note that PR is expressed in recurrence (rec.) therapy and prevention group. Scale bar, 50 μm. (**B**) ERα expression is similar in all cancers. Seven cervical cancers per group were stained for ERα (green) and representative images are shown. Nuclei are shown in blue. A cervical tissue section from *Esr1*^−/−^ (ERαKO) was used as negative control. Dotted lines separate cervical cancer (cc) or epithelium (ep) from stroma (st). Scale bar, 50 μm. (**C**) MCM7 expression is similar in all cancers. Seven cervical cancers per group were stained for MCM7 (red), marker for E7 function and representative images are shown. Nuclei are shown in blue. Normal mouse IgG was used as negative control. Dotted lines separate cervical cancer (cc) from surrounding stroma (st). Scale bar, 30 μm.

The main target of MPA is PR [25]. As previously reported [23], cervical cancers in the primary group expressed PR (Figure 5A). PR expression was also evident in cervical cancers not only in the recurrence (+E_2_) and recurrence (−E_2_) but also in recurrence therapy and prevention group (Figure 5A). One of seven mice in the prevention group had cervical cancer that displayed reduced PR expression compared to the proximal epithelium and surrounding stroma as well as other cancers (Supplementary Figure S3). While the incidence of PR−positive cancer was not significantly different among all groups (*P* ≥ 0.5), it raised a possibility that PR loss may be responsible for a subset of recurring cervical cancers. Nonetheless, these results indicate that the loss of PR expression is not the major mechanism of recurrence and MPA resistance. ERα is required for cervical carcinogenesis in the HPV transgenic mouse model [18]. Cervical cancers in all groups expressed ERα (Figure 5B). Expression of MCM7, cervical cancer biomarker [16], was similar in all cancers (Figure 5C).

## DISCUSSION

MPA promotes regression of primary cervical cancer in the HPV transgenic mouse models [23]. Our results indicate that MPA fails to completely eliminate cancer cells because cervical cancer recurred at high frequency (100%) after MPA therapy (Table 1). Cervical cancer recurs after raloxifene therapy in the HPV transgenic mouse model; however, the incidence of cancer recurrence in the presence or absence of exogenous E_2_ is 72.8% (*n* = 11) and 40% (*n* = 10), respectively [26]. The lower rate of cancer recurrence suggests that inhibition of ERα by raloxifene may be more effective in treating primary cervical cancer than activating PR by MPA. It remains to be determined whether combination therapy with raloxifene and MPA results in no cancer recurrence.

The development of primary cervical cancer in the HPV transgenic mice requires exogenous E_2_ [15, 27]. Cervical cancer recurred after MPA therapy even in the absence of exogenous E_2_ (Table 1). These observations highlight the difference between primary and recurrent cervical cancer. Although cervical cancer recurs in the absence of exogenous E_2_ after raloxifene therapy, the recurrence depends on endogenous E_2_ [26]. While primary cervical cancer grows better in the presence of exogenous E_2_, endogenous E_2_ also contributes to the persistence of the primary cancer [28, 29]. These observations suggest that both exogenous and endogenous E_2_ contribute to development of primary and recurrent cervical cancer. Exogenous E_2_ did not increase incidence or multiplicity of recurrent cervical cancer, but resulted in larger recurrent cancer (Figure 2A–2C). However, proliferation and apoptosis indices of recurrent cancers were similar in the presence or absence of exogenous E_2_ (Figure 2D–2G). All mice were sacrificed 2 months after MPA therapy. Thus, we postulate that, in the presence of exogenous E_2_, the cancer recurred earlier and thus had grown for longer period of time compared to the cancer recurred without exogenous E_2_.

It is notable that 4 of 16 mice in the De Novo group had cervical cancer (Table 1). These mice were treated with exogenous E_2_ only for 2 months starting at 9−9.5 months of age (Figure 1A). Mice expressing E6 and E7 do not succumb to cervical cancer when treated with exogenous E_2_ for 3 months starting at 1−1.5 month of age [27, 29]. Cervical cancer is also observed in *K14E6/K14E7* double transgenic mice treated with exogenous E_2_ for 3 months starting at 8−8.5 months of age after raloxifene therapy [26]. These observations suggest that older mice are more susceptible to cervical carcinogenesis. Perhaps, cervical neoplastic disease develops spontaneously in some mice as they age, and exogenous E_2_ accelerates disease progression.

Cancers in the recurrence therapy group were significantly smaller (> 2−fold) than the control group (Figure 4B–4C). The proliferation index of the recurrence therapy group was reduced only by 30% compared to the control group (Figure 4E). Thus, difference in proliferation does not fully account for the smaller cancer size in the recurrence therapy group. Differentiated epithelial cells are larger than undifferentiated cells [19]. Poorly differentiated cancers in the recurrence therapy group (Figure 3B–3C) may contribute to the difference in cancer size. Nonetheless, the fact that MPA decreased proliferation of recurrent cervical cancer raises a possibility that the recurrent cancer may fully regress if MPA treatment is prolonged. While we could not test possibility due to high morbidity of the mice older than the recurrence therapy group, cervical cancer recurred even in the presence of MPA (Table 1; prevention group). The morbidity was not related to cervical cancer [26, 30]. Therefore, we believe that the cancer would not disappear even if recurrent cervical cancer were treated with MPA for longer than 2 months. These results strongly support that recurrent cervical cancer is resistant to MPA. Perhaps, MPA–sensitive and –resistant cells are present in cervical cancer recurred in the absence of MPA. Our results indicated that PR was required for MPA's therapeutic effect on cervical cancer (F.F. Mehta, S. Baik and S.H. Chung, unpublished data). The loss of PR expression is not the main mechanism of resistance to MPA (Figure 5A). It is possible that PR downstream signaling pathways responsible for anti−tumor activity are disrupted in the recurrent cervical cancer. MPA is used to treat early stage of endometrial cancer that expresses PR. However, most endometrial cancers eventually lose PR expression and stop responding to MPA [31, 32]. Interestingly, some PR−positive endometrial cancers do not respond to MPA therapy, and its mechanism remains to be determined [31]. PR is expressed in 20−40% of human cervical cancer [21, 33–35]. If our results are translatable to women, some of patients with PR−positive cervical cancer may initially benefit from MPA therapy, but recurrence of MPA−resistant cancer will be anticipated.

Our results demonstrated that cervical cancer recurred at high frequency after MPA therapy and the recurring disease was not so responsive to the same therapy as primary cancer. While the results are discouraging with regard to the translational value, they provide a model system to study mechanisms of recurrence and therapy resistance of cervical cancer. Such mechanisms may be relevant to resistance of endometrial cancer to MPA. Further studies to identify PR target genes and pathways that mediate anti-cervical cancer activity of MPA are warranted.

## MATERIALS AND METHODS

### Transgenic mice and treatments

The *K14E6* and *K14E7* transgenic mice have been described previously [13, 14]. *K14E6/K14E7* double transgenic mice were generated by mating *K14E7* hemizygote males with *K14E6* homozygous females. Mice were genotyped by PCR. Four to six week old *K14E6/K14E7* virgin females were treated with slow−release 17β−estradiol (E_2_) tablets (0.05 mg/60 days; Innovative Research of America, Sarasota, FL) as previously described [15]. Some mice were also subjected to monthly i.p. injection of MPA Injectable Suspension (SICOR Pharmaceuticals, Irvine, CA), which delivers 4.5 mg of MPA for a month [23]. All mice were i.p. injected with bromo−deoxyuridine (BrdU; 3.75 mg/mouse) 1 hour before collecting tissues. All procedures were approved by the University of Houston Institutional Animal Care and Use Committee.

### Tissue processing and histopathological analysis

Reproductive tracts were fixed in 4% paraformaldehyde, embedded in paraffin, and serially sectioned at 5 μm thickness throughout the cervix. Hematoxylin and eosin (H&E) staining was carried out as previously described [23]. Every tenth slide was subjected to blinded histopathological analyses as previously described [15].

### Immunohistochemistry and TUNEL assay

For immunohistochemistry (IHC), sections were deparaffinized, rehydrated, and microwaved in 10 mM sodium citrate buffer (pH 6.0) for 20 minutes. After incubation with blocking buffer (10% goat serum in PBS), the sections were incubated with primary antibodies as previously described [19, 36]. Anti−K14 antibody (BioLegend, San Diego, CA) was diluted to 10% goat serum (1:1,000). The sections were subsequently incubated with secondary antibody conjugated with Alexa488 or 594 (Life Technologies). TUNEL assay was carried out using ApopTag Fluorescein *in situ* apoptosis detection kit according to the manufacturer's instructions (Millipore). Nuclei were stained with Hoechst 33342.

### Microscopy and digital image analyses

Stained tissue sections were visualized by an Olympus BX51 microscope. Representative images were acquired with color (Olympus DP73) or cooled CCD monochrome cameras (Olympus XM10). Measurement of tumor size was carried out using the Olympus cellSens Dimension imaging software on images acquired with a 20X objective lens as previously described [28]. For quantification of BrdU− and TUNEL−positive cells, several random microscopic fields per cancer were analyzed.

### Statistical analyses

All statistical analyses were carried out using the MSTAT software (version 6.1.4), which is freely available at mcardle.wisc.edu/mstat. Cancer incidence was analyzed using one−sided Fisher's exact test. One−sided Wilcoxon rank sum test was used for proliferative and apoptotic indices, multiplicity, and cancer size.

## SUPPLEMENTARY MATERIALS FIGURES AND TABLES


