# Supplementary material for: Recurrence of cervical cancer and its resistance to progestin therapy in a mouse model
Source: Oncotarget. 2016 Nov 29;8(2):2372–80. doi: 10.18632/oncotarget.13676 (PMC5356807; doi:10.18632/oncotarget.13676)
Supplement: Supplementary file 1 [file oncotarget-08-2372-s001.pdf]

Recurrence of cervical cancer and its resistance to progestin therapy in a mouse model

Supplementary Materials

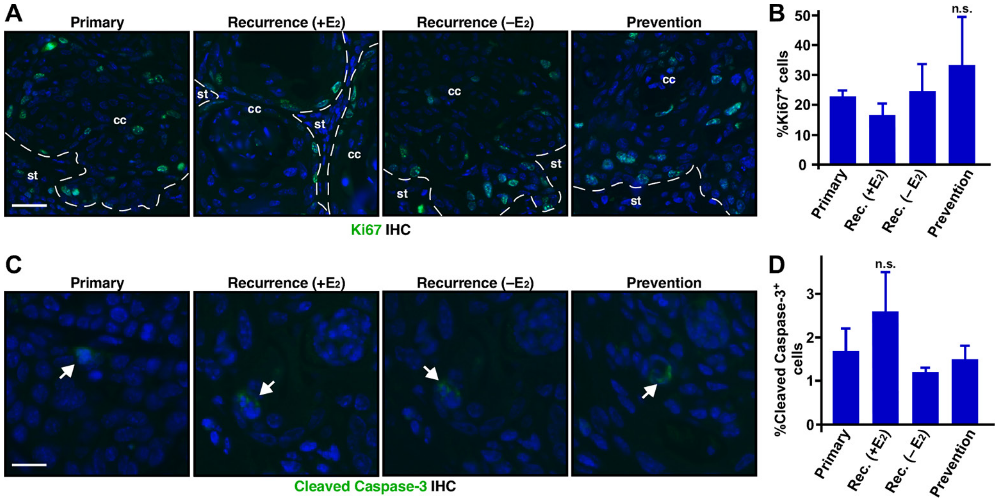

Supplementary Figure S1: E_2 and MPA do not influence expression of Ki67 and cleaved caspase-3 during recurrence. (**A**) Cervical cancer sections were stained for proliferation marker Ki67 (green). Nuclei are shown in blue. Dotted lines separate cervical cancer (cc) from stroma (st). Scale bar, 30 µm. (**B**) Results shown in A. was quantified and shown as mean ± S.E.M. (n = 3). n.s., not significant. (**C**) Cervical cancer sections were stained for apoptosis marker cleaved caspase-3 (green). Nuclei are shown in blue. Scale bar, 15 µm. (**D**) Results shown in C. was quantified and shown as mean ± S.E.M. (n = 3). n.s., not significant.

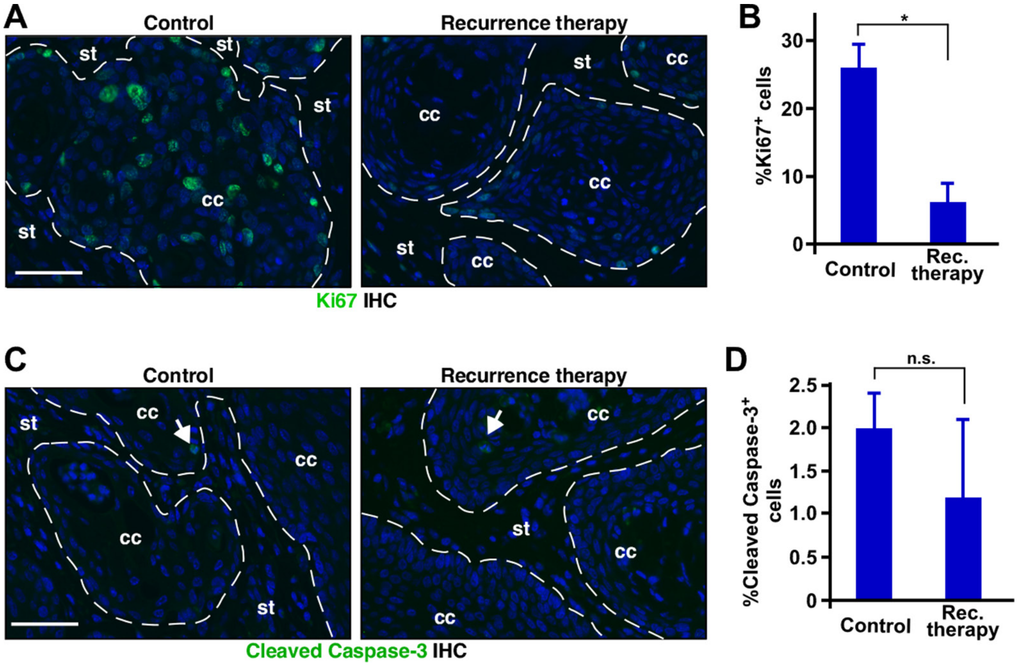

Supplementary Figure S2: MPA decreases expression of Ki67 in recurrent cervical cancer. (**A**) Cervical cancer sections were stained for proliferation marker Ki67 (green). Nuclei are shown in blue. Dotted lines separate cervical cancer (cc) from stroma (st). Scale bar, 50 μm. (**B**) Results shown in A. was quantified and shown as mean ± S.E.M. ($n = 3$). $P = 0.05$. (**C**) Cervical cancer sections were stained for apoptosis marker cleaved caspase-3 (green). Nuclei are shown in blue. Dotted lines separate cervical cancer (cc) from stroma (st). Scale bar, 50 μm. (**D**) Results shown in C. was quantified and shown as mean ± S.E.M. ($n = 3$). n.s., not significant.

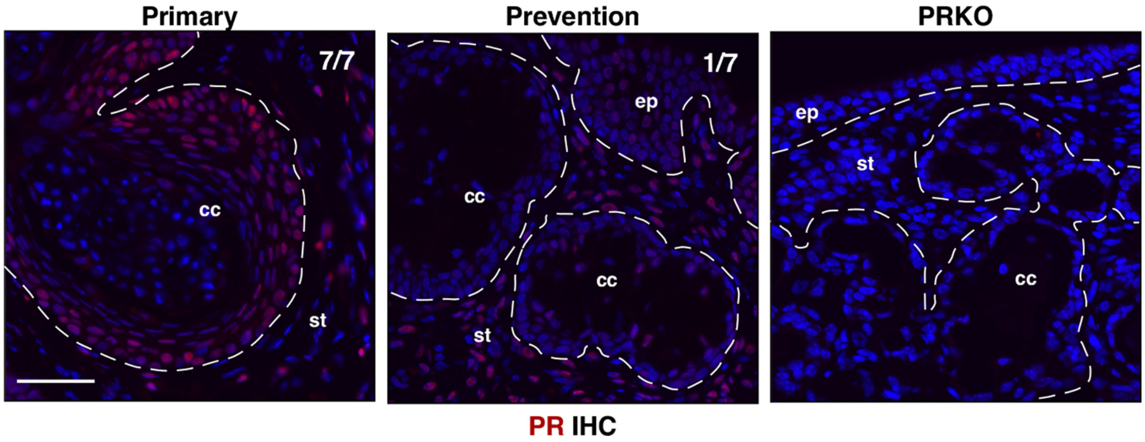

Supplementary Figure S3: PR expression is reduced in one cervical cancer recurred in the presence of MPA. Seven cervical cancers per group were stained for PR (red) as in Figure 5A. Note that reduced PR expression was observed in 1 of 7 cancers we analyzed in the prevention group. Dotted lines separate cervical cancer (cc) from stroma (st). PRKO is *K14E7/Pgr*$^{-/-}$. Scale bar, 50 μm.
